# Piezoelectricity and rotostriction through polar and non-polar coupled instabilities in bismuth-based piezoceramics

**DOI:** 10.1038/srep28742

**Published:** 2016-07-01

**Authors:** Matias Acosta, Ljubomira A. Schmitt, Claudio Cazorla, Andrew Studer, Alexander Zintler, Julia Glaum, Hans-Joachim Kleebe, Wolfgang Donner, Mark Hoffman, Jürgen Rödel, Manuel Hinterstein

**Affiliations:** 1 Department of Geo- and Materials Science, Technische Universität Darmstadt, Alarich-Weiss-Strasse 2, 64287 Darmstadt, Germany; 2 School of Materials Science and Engineering, UNSW Australia, Sydney, New South Wales 2052, Australia; 3 Bragg Institute, Australian Nuclear Science and Technology Organization, Locked Bag 2001, Kirrawee DC NSW 2232, Australia; 4 Department of Materials Science and Engineering, Norwegian University of Science and Technology, Trondheim, 7491, Norway; 5 Institute for Applied Materials, Karlsruhe Institute for Technology, P.O. Box 3640, 76021 Karlsruhe, Germany

## Abstract

Coupling of order parameters provides a means to tune functionality in advanced materials including multiferroics, superconductors, and ionic conductors. We demonstrate that the response of a frustrated ferroelectric state leads to coupling between order parameters under electric field depending on grain orientation. The strain of grains oriented along a specific crystallographic direction, 〈h00〉, is caused by converse piezoelectricity originating from a ferrodistortive tetragonal phase. For 〈hhh〉 oriented grains, the strain results from converse piezoelectricity and rotostriction, as indicated by an antiferrodistortive instability that promotes octahedral tilting in a rhombohedral phase. Both strain mechanisms combined lead to a colossal local strain of (2.4 ± 0.1) % and indicate coupling between oxygen octahedral tilting and polarization, here termed “rotopolarization”. These findings were confirmed with electromechanical experiments, *in situ* neutron diffraction, and *in situ* transmission electron microscopy in 0.75Bi_1/2_Na_1/2_TiO_3_-0.25SrTiO_3_. This work demonstrates that polar and non-polar instabilities can cooperate to provide colossal functional responses.

Multifunctional ceramics define breakthrough technologies due to their unique ability of transducing electrical, mechanical, optical, and magnetic signals^1^. This is a consequence of the rich and complex interplay between their order parameters and conjugated fields^2^. In ferroelectric and ferroelastic materials, for instance, polarization and strain are typical order parameters that can be controlled through the application of an electric field and stress, respectively (see Fig. 1). The development of polarization and its reorientation with electric field are considered fingerprints of ferroelectricity (FE) and are ascribed to the softening of a zone-center *polar* phonon mode in the reference centrosymmetric phase the electromechanical response generally considered in ferroelectrics is piezoelectricity and electrostriction, which indicate that the strain and polarization are coupled order parameters (Fig. 1(1)). Another order parameter in perovskites is provided by the oxygen octahedral tilts *φ*. These tilts occur due to a structural instability in the reference centrosymmetric configuration that involves the softening of a *non-polar* zone-boundary vibrational mode and are termed antiferrodistortive (AFD). The development of octahedral tilts leading to strain, which indicates their coupling, is allowed by fundamental crystal symmetry arguments and is termed rotostriction (Fig. 1(2))^3^. The coupling or combined action of piezoelectricity and rotostriction, however, has remained mostly elusive.

FE and AFD instabilities in general compete with each other. Cases in which both distortions are mutually coupled are rare (Fig. 1(3))^4^,^5^,^6^,^7^,^8^,^9^,^10^. Research on superlattices has revealed remarkable examples where octahedral tilts can strongly enhance or even induce a ferroelectric distortion in materials that would otherwise be non-polar^11^,^12^. Theoretical studies have shed light on ways to manipulate and design FE-AFD couplings that could potentially trigger unprecedented functionalities in new perovskite materials via external perturbations^11^,^12^,^13^,^14^,^15^,^16^,^17^,^18^, leading to the discovery of new functionalities in engineered thin films^19^. FE and AFD instabilities are present in a large number of engineered thin films^20^ and bulk perovskites^10^, including the technologically most relevant Pb(Zr,Ti)O_3_ (PZT)^6^,^7^,^8^,^9^. Nevertheless, experimental evidence on the direct control of FE-AFD couplings is lacking to date.

Bi_1/2_Na_1/2_TiO_3_ (BNT)-based materials are prime candidates in the quest for lead-free environmentally friendly alternatives to PZT due to their giant strain response^21^,^22^,^23^. These materials feature phases with FE and AFD instabilities, attributed to a frustrated ferroelectric state inherent to their local disorder^24^. For this study, we selected the (1−x)Bi_1/2_Na_1/2_TiO_3_-xSrTiO_3_ system with 25 mol % SrTiO_3_ (BNT-25ST) since it features a core-shell structure with local polar and non-polar phases representing different octahedral tilts^22^. This intricate microstructure entails a strong competition between FE and AFD instabilities and is a prominent candidate to investigate FE-AFD couplings.

Here we investigate the relationship between order parameters in BNT-25ST when subjected to an electric field. Electromechanical characterization and *in situ* neutron diffraction were performed to characterize the average macroscopic strain and structural distortions including the octahedral tilts. *In situ* neutron diffraction experiments were done at several orientations of the sample with respect to the electric field allowing to investigate orientation-dependent average structural distortions. *In situ* transmission electron microscopy (TEM) was also employed to investigate local structural distortions. Our experimental assessments clarify the interplay between FE and AFD distortions in this material with the finding that coupling between polar and non-polar instabilities leads to a colossal local strain. Our interpretations of the experimental observations are underpinned by a phenomenological Landau-Ginsburg-Devonshire free energy model.

## Results and Discussions

### Macroscopic Characterization

The macroscopic strain response of BNT-25ST as a function of time is introduced in Fig. 2(a) for electric field pulses varying between 1.5 kV/mm and 4 kV/mm. The change in strain from 1.5 kV/mm to 2 kV/mm (pointed out with a dashed arrow) is around 0.12% and corresponds to an increment of 65% in the strain response of the material with 0.5 kV/mm; i.e., from 1.5 kV/mm to 2 kV/mm. Further enhancements of the probing electric field lead to much more gradual increments in strain below 15% for each step. The considerably high change in strain from 1.5 kV/mm to 2 kV/mm suggests an electric field induced phase transformation into a ferroelectric state that should be followed by domain switching^22^,^25^,^26^. The microstructure of BNT-25ST consists of cores that transform irreversibly from a non-ergodic relaxor state into a ferroelectric state, whereas the surrounding shells transform reversibly from an ergodic relaxor state into a ferroelectric state providing the bulk strain response^22^. We note here that only the average macroscopic electric field for the phase transformation was assessed. However, microscopically the values required for the phase transformation of core and shell may differ considerably due to the relative permittivity of the phases. The inset of Fig. 2(a) depicts the decay in strain upon electric field removal. The lower remanent strain measured at 1.5 kV/mm as compared to the remanent strain at other field strengths suggests that the threshold field for the irreversible phase transformation of cores lies between 1.5 kV/mm and 2 kV/mm. Thereafter, for higher electric field the irreversible transformation of cores leads to a higher remanent strain. The remanent strain observed after 20 s of electric field removal is below 10% of the values of the strain under electric field, indicating reversibility of field induced processes in the shell regions^22^.

*In situ* orientation-dependent neutron diffraction experiments were employed for quantifying the average structure evolution of BNT-25ST under electric field. Figures 2(b,c) introduce contour plots of the ½ 311, 111, and 200 reflections (indexing refers to the primitive cubic aristotype) for the remanent state at *E* = 0 kV/mm (after electric field removal) and during field application of *E* = 3 kV/mm. The virgin state mimics the remanent state and is not shown. The white lines superimposed on the images outline the two extreme cases where the scattering vectors *k*_*hkl*_ are parallel or orthogonal to *E*. These lines exhibit a slope of ½ as *k*‖*E* only occurs for *ω* = *θ*. Since a diffraction pattern of polycrystalline materials is composed of intensities of randomly oriented diffracting grains that fulfil the Bragg condition, grains contributing to ½ 311, 111, and 200 reflections do not necessarily coincide. From now on we will refer to the scattering vectors of the main reflections simply as *k*, since *k* expresses the crystallographic grain orientation with respect to the electric field.

In the remanent state displayed in Fig. 2(b), the main reflections 111 and 200 are characterized by orientation-independent intensities and positions. Moreover, no splitting of main reflections is evidenced. These features suggest an average phase with few non-cubic distortions and limited remanent lattice strain upon electric field removal. Even though the intensity is plotted on a logarithmic scale, the ½ 311 superstructure reflections (SSR) cannot be distinguished from the background. The features of the ½ 311, 111, and 200 reflections in the remanent state indicate that the electric field induced processes, described in the subsequent paragraphs, are almost entirely reversible supporting the low remanent state depicted in Fig. 2(a). The virgin and remanent states of BNT-25ST are illustrated schematically by the two pseudocubic grains introduced in Fig. 2(d).

Under *E* = 3 kV/mm, the ½ 311, 111, and 200 reflection positions and intensities vary significantly. The presence of ½ 311 SSR suggests a rhombohedral phase with *R3c* symmetry characterized by anti-phase (a^−^a^−^a^−^) (Glazer notation^27^) octahedral tilting and polarization along the [111] axis. The maximum SSR intensity is featured at *ω* ~− 15° (marked with an arrow). The condition of *ω* ~− 15° lies between *k*∥*E* and *k*⊥*E*, which reflects the spatial orientations of the 〈311〉 direction with respect to the 〈*h*00〉 and 〈*hhh*〉 directions. The 200 reflection exhibits pronounced splitting in the ω range between −15° and 60° indicating a second phase of tetragonal character. Since no ½ *ooe* SSR (where *o* stands for odd and *e* for even indices) are visible in the complete diffractogram, the most probable tetragonal symmetry is *P*4*mm* with polarization along [001]. We note, however, that we cannot discard local distortions with *P4bm* symmetry^28^,^29^. Any other orientation with respect to the electric field reveals an intermediate shift of main reflections and intermediate intensity of SSR. Note that the variation of the intensity of ½ 311 SSR cannot be ascribed to texturing, since this reflection does not allow splitting.

**Figure 1:**
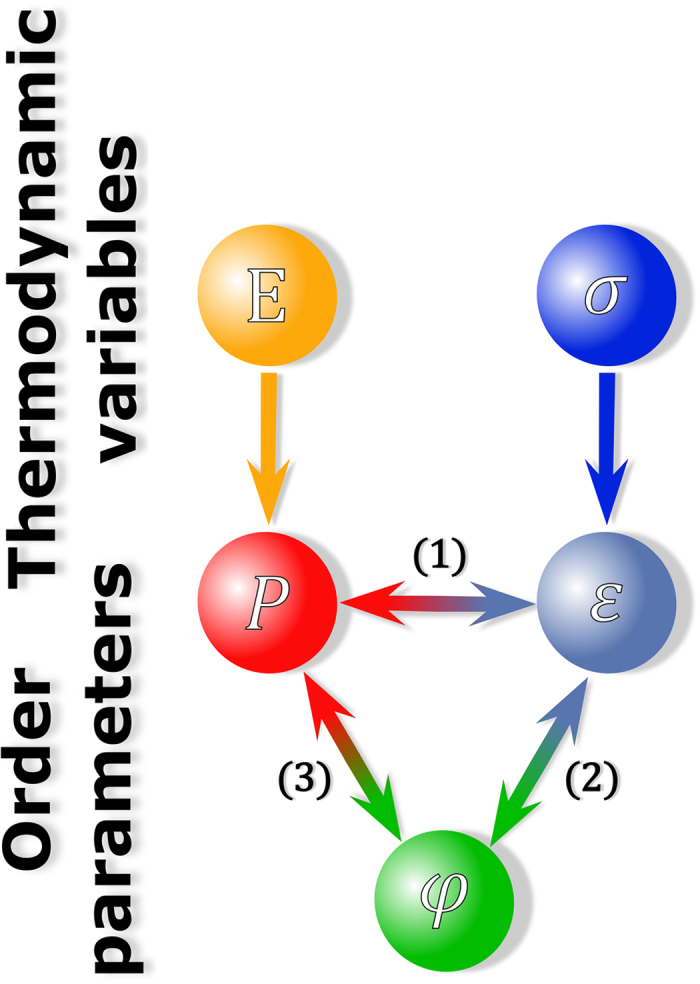
Thermodynamic variables electric field *E* and stress *σ* are conjugated to the order parameters polarization *P* and strain *ε*, respectively. Coupling between the order parameters polarization *P* and strain *ε* is termed piezoelectricity/electrostriction (1), between strain *ε* and oxygen ochtaedral tilts *ϕ* is termed rotostriction (2), and between polarization *P* and oxygen ochtaedral tilts *ϕ* is termed in this work as rotopolarization (3).

**Figure 2:**
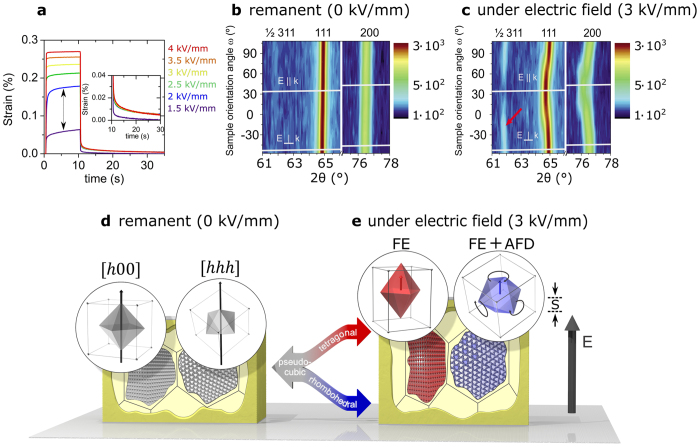
Macroscopic strain mechanisms. (**a**) Strain as a function of time for pulses of 10 s duration and electric fields ranging between 1.5 kV/mm and 4 kV/mm. Inset of (**a**) displays the strain decay after electric field removal. (**b**) remanent state at 0 kV/mm and (**c**) applied electric field state at 3 kV/mm contour plots of *in situ* neutron diffraction patterns for sample orientations in the ω range between −57° and 108°. The presented 2θ angular range of interest corresponds to the ½ 311, 111, and 200 reflections. The white lines superimposed indicate the patterns at ω = θ and ω = θ −90° which fulfil the condition of *k*∥*E* and *k*⊥*E*, respectively. The red arrow indicates the maximum intensity of the ½ 311 reflection. (**d**,**e**) visualize the two ideal cases of grains with 〈*h*00〉 and 〈*hhh*〉 oriented along the electric field. The strain of the material under electric field is given by *S*. Pseudocubic grains in the virgin state (grey) transform to either tetragonal (red) or rhombohedral (blue) symmetry due to FE or AFD instabilities.

For the case of *k*∥*E*, the maximized shift in position of the main reflections indicates a strong electric field induced lattice strain and thus polarization. This is a consequence of the selective view along the macroscopic expansion direction. Moreover, the intensity of the ½ 311 SSR remains negligible, similar to the virgin and remanent states. Therefore, the grains that satisfy *k*∥*E* transform predominantly to the *P4mm* tetragonal phase since this phase prohibits octahedral tilting. The latter can be related to 〈311〉 being nearer in space to 〈*h*00〉, rather than to 〈*hhh*〉. The maximized lattice strain is thus promoted due to a FE distortion, indicating that polarization and strain are coupled. Therefore, grains satisfying *k*∥*E* feature the maximized converse piezoelectric effect and electrostriction (Fig. 1(1)). This strain mechanism is schematically depicted by the left grain of Fig. 2(e).

For the case of *k*⊥*E*, the main reflections are shifted to higher diffraction angles indicating macroscopic shrinkage perpendicular to the electric field direction as compared to the remanent state. Meanwhile, the ½ 311 SSR displays non-zero intensity, indicating that, upon the application of electric field, the development of an average lattice distortion and thus polarization is also accompanied by the appearance of octahedral tilts. This entails the conjugation between polarization and octahedral tilts, which is only observed for the rhombohedral phase with *R*3*c* symmetry since no tetragonal SSR develop for the *P4mm* tetragonal phase. Therefore, the strain response of the grains that transform to the *R*3*c* rhombohedral phase when subjected to an electric field is a result of both, converse piezoelectric effect/electrostriction (Fig. 1(1)) and rotostriction (Fig. 1(2)). This strain mechanism is schematically depicted in the right grain of Fig. 2(e). A reorientable polarization is a general property of ferroelectrics with a FE distortion, whereas octahedral tilts are characteristic for materials with an AFD distortion. This entails that Fig. 2(e) is a schematic representation of a FE-AFD coupling in the grains of BNT-25ST that transform to the rhombohedral phase (Fig. 1(3)). From here on, we termed the FE-AFD coupling; *i.e.*, coupling between polarization and oxygen octahedral tilting, as rotopolarization.

### Phenomenological Model

The relations between the order parameter couplings described above can be rationalized in terms of a Landau-Ginsburg-Devonshire phenomenological model^30^. Consider the following expansion of the elastic Gibbs free energy, Δ*G*, expressed as a function of polarization *P*_*i*_, oxygen octahedral tilt *φ*_*i*_, and stress *σ*_*i*_ (Voigt notation is considered):


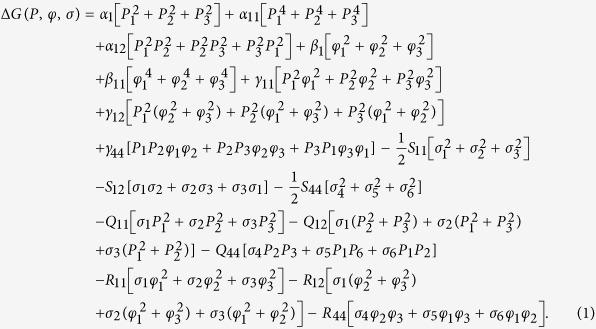


The coefficients of this energy function are: *α* dielectric stiffness, *β* octahedral torsion coefficients, *γ* coupling between ferroelectric polarization and oxygen tilt angle, *S* elastic compliances, *Q* electrostrictive coupling coefficients, and *R* rotostrictive coupling coefficients.

The following two solutions to the energy function (Eq. 1) are of interest: (1) ferroelectric tetragonal with *P*_1_ = *P*_2_ = 0 and *P*_3_ ≠ 0 and *ϕ*_1_ = *ϕ*_2_ = *ϕ*_3_ = 0; and ferroelectric rhombohedral with *P*_1_ = *P*_2_ = *P*_3_ ≠ 0 and *ϕ*_1_ = *ϕ*_2_ = *ϕ*_3_ ≠ 0. Since BNT-25ST features a frustrated ferroelectric state, α_1_ and *β*_1_ in Eq. 1 must be equal to zero by construction to fulfil the requirement that at zero electric field the spontaneous polarization and octahedral tilts are null (as on average in a pseudocubic structure such as observed in Fig. 2(b)). It can be shown that under conditions of zero stress, the spontaneous elastic strain, *ε* = (∂*G*)/(∂*σ*), along the direction of the electric field in the tetragonal (T) and rhombohedral (R) phases are given by Eqs 2 and 3, respectively.


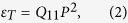



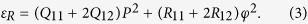


Hence, strain of grains that transform to the tetragonal phase is not coupled to the oxygen octahedral tilts, in contrast to strain of the grains that transform to the rhombohedral phase.

In order to establish a quantitative description of the experimentally demonstrated FE-AFD coupling, let us assume elastic coherence between grains transforming to tetragonal and rhombohedral phases in BNT-25ST (*i.e., ε*_*T*_ ≈ *ε*_*R*_), as provided in Eq. 4.


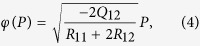


where *Q*_12_ < 0.

Equation 4 provides an approximate quantitative expression that describes the coupling between polarization and octahedral tilts in BNT-25ST. We note, however, that this expression neglects the residual stresses commonly found in ferroelectrics that may account up to 0.2%^31^. Equation 4 indicates that with the assumption of elastic coherence, the octahedral tilts and polarization are coupled via electrostrictive and rotostrictive coefficients. The coexistence of grains with tetragonal phase and rhombohedral phase with octahedral tilting guarantee a coherent elastic response. Such concept should not be misinterpreted by considering that the elastic coherence assumed in the descriptive model is responsible for allowing the simultaneous appearance of polarization and octahedral tilts. Therefore, the experimental results displayed in Fig. 2(c) and the descriptive phenomenological model presented here support the existence of a genuine FE-AFD coupling in BNT-25ST, *i.e.*, suggest the existence of rotopolarization (Fig. 1(3)).

### Microscopic Characterization

**Figure 3:**
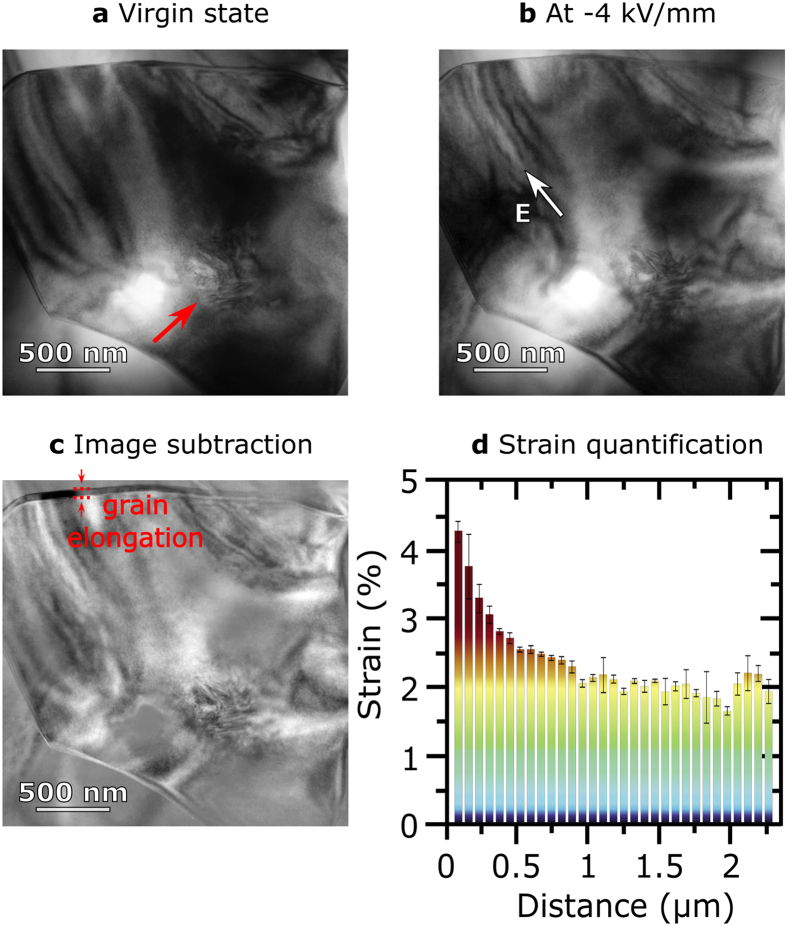
Strain quantification via TEM. TEM bright field images of a grain viewed along pseudocubic <111> zone axis in (**a**) initial state with presence of core (marked with red arrow) and shell and (**b**) at −4 kV/mm. (**c**) image subtraction between the grain at 0 kV/mm and −4 kV/mm. In the merged image an elongation of the grain is visible along the upper part of the figure, as indicated. (**d**) local strain quantification with a mean strain value of (2.4 ± 0.1) %.

So far we only probed the electromechanical response and orientation-dependent distortions of BNT-25ST on average. Thus the converse piezoelectric effect/electrostriction and rotostriction were probed with intergranular constraints. This does not allow to identify the combined piezoelectric and rotostrictive effects in the grains that transform to a rhombohedral phase (right grain of Fig. 2(e)). For this purpose, it is mandatory to probe either a properly oriented quasi-free standing rhombohedral grain or a single crystal. Single crystals of BNT-25ST have not been synthesized so far. Hence, we focus on the local investigation of a grain by means of *in situ* TEM. Figure 3(a) introduces the virgin state of a grain with a core-shell structure viewed along the [111]_pc_ zone axis. The representative grain consists of a ferroelectric core with presence of domains (marked with red arrow) and a homogeneous shell, where no visible domain structure is present. Figure 3(b) displays the same grain with an applied electric field along the [0−11]_pc_ direction, fulfilling the previously discussed condition of *k*⊥*E* leading to piezoelectricity and rotostriction. It is apparent that application of the electric field leads to changes in the domain-like contrast, as well as an elongation of the grain. In order to elucidate the grain elongation, image subtraction between the virgin state and that under electric field was performed (Fig. 3(c)). The elongation of the grain is visualized by a change in contrast along the complete grain, as indicated. Apart from elongation, other changes in the contrast of Fig. 3(c) may be attributed to changes in the grain dimensions parallel to the electron beam or changes in domain-like contrast.

Figure 3(d) introduces the strain quantification of the selected grain from left to right. It is observed that the grain strains between (1.7 ± 0.1) % and (4.4 ± 0.2) %. The non-uniform strain may be attributed to the local strain fields surrounding the grain and the local electric field distribution. The mean strain value of the grain accounts for a colossal strain output of (2.4 ± 0.1) %, due to both piezoelectricity and rotostriction.

The deformation of a quasi-free standing grain; i.e., a grain in a thin foil such as investigated by TEM in this work, resembles the expected electromechanical response of a single crystal. The mean colossal strain output of (2.4 ± 0.1) % at 4 kV/mm suggests that a single crystal of BNT-25ST may feature a normalized strain output of 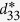 ≈ 6000 pm/V at 4 kV/mm. Lead-containing single crystals with 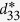 ≈ 2500 pm/V at 6 kV/mm^32^ and lead-free single crystals with 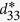 ≈ 3000 pm/V at 2 kV/mm were reported^33^. Nonetheless, a colossal strain such as found in BNT-25ST in this work, has not been realized in any piezoelectric^34^. This indicates that a properly oriented BNT-25ST single crystal may constitute a breakthrough of significant technological relevance for actuator applications. We note, however, that properly synthesized single crystals may not feature a core-shell microstructure, thus, the extrapolation of this mechanism to single phase crystals and other polycrystalline materials warrants further experimental proof.

In contrast to other BNT-based materials^35^, BNT-25ST displays an electric field induced orientation-dependent phase transformation into a long range ferroelectric (polar) distorted order that consists of a mixture of tetragonal and rhombohedral phases. The reversible phase transformation is mostly attributed to the response of the shell^22^ and allows exploitation of the FE and AFD instabilities at each field cycle. The core-shell structure of BNT-25ST appears pertinent to the strain mechanisms since, apart from a redistribution of the local electric field, it may impart an initial state with two different phases that feature different polarization and oxygen octahedral tilts^22^. This promotes a strong competition between FE and AFD instabilities and, together with the different grain orientations with respect to the electric field, favors the nucleation of either the average rhombohedral or tetragonal phases. The orientation-dependent phase nucleation should favor the overall electromechanical response of the system since it may help to maximize the achievable strain of preferential crystallographic orientations^36^.

The rhombohedral phase of BNT-25ST is especially relevant since it features a polar structural distortion and octahedral tilts. This allows to control, to a certain extent, the magnitude of the oxygen octahedral tilts via the application of an electric field. This behavior departs significantly from what one would expect in perovskites, where polarization and octahedral tilt are considered competing instabilities that suppress each other^4^. The FE-AFD coupling of BNT-25ST appears to extend the rotostrictive coupling observed at superstructures, previously described as an interface effect^11^, to bulk materials thus leading to the existence of rotopolarization. This renders an alternative strategy to maximize electromechanical properties of piezoelectrics other than the search of interferroelectric phase boundaries^37^,^38^ or critical points^39^.

Future research of FE-AFD couplings is foreseen in materials with tailored microstructures with competing order parameters such as the core-shell of BNT-25ST or heterostructures^19^,^20^,^40^, or where coexistence of FE and AFD phases is encountered. The latter may be in fact found in PZT at the composition-temperature coordinates where the low and high temperature rhombohedral phases meet the morphotropic phase boundary.

The potential of colossal functional responses as a result of FE and AFD couplings is not limited to tailor the electromechanical properties of ferroelectrics. Gopalan and Litvin^3^ developed symmetry operations for FE and AFD distortions in perovskites. Although coupling between these distortions was not realized, it was clearly demonstrated that they can both affect electric, magnetic, and optical properties. Therefore, the search of FE and AFD couplings for tailoring the functionality of perovskites opens a new avenue for the next generation of multifunctional materials.

## Conclusions

The intricate interplay between structural instabilities and their dependencies on application of external perturbations is mandatory for the rational engineering of properties in multifunctional materials. In order to maximize, for instance, the strain output of ferroelectrics, these materials have been traditionally designed around ferrodistortive phase instabilities. In this work, however, we demonstrate that, counter to intuition, antiferrodistortive instabilities can also contribute synergetically to the strain output. In fact, we clarify that the joint effect of ferrodistortive and antiferrodistortive structural instabilities can develop a colossal local strain response of (2.4 ± 0.1) %.

In view of our results, we propose that the search of couplings between polar and non-polar instabilities, like the one described in this work, can lead to the design of complex ceramics with improved functionality. We encourage the search of these types of couplings near frustrated states, engineered microstructures, or at temperature-compositional regions where ferrodistortive and antiferrodistortive phases coexist. The present study opens a new avenue for future research and development of the next generation of multifunctional materials.

## Material and Methods

Ceramics were produced via a mixed oxide route using reagent grade oxides and carbonates Bi_2_O_3_ (99.975%), Na_2_CO_3_ (99.5%), TiO_2_ (99.9%), and SrCO_3_ (99%) (Alfa Aesar GmbH & Co. KG, Germany). Powders were mixed according to the stoichiometric formula 0.75Bi_1/2_Na_1/2_TiO_3_-0.25SrTiO_3_. Complete processing details can be found elsewhere^26^.

Room temperature strain measurements were performed in a silicon oil bath with an optical displacement sensor (Philtec Inc, USA). The input signal chosen was a rectangular shape of 10 seconds duration and amplitude ranging from 1.5 to 4 kV/mm. Considering the finite time response of domain switching^41^, pulse experiments were performed to elucidate the phase transition with minimized influence from domain switching. A direct voltage source Voltcraft DIGI 40 (Rapid electronics Ltd, UK) was used to charge a home-made fast switching device (150 ns discharge time) based on a fast high voltage transistor switch HTS-41-03-GSM (Behlke Power Electronics GmbH, Germany).

Neutron diffraction studies were performed at the high-intensity diffractometer Wombat^42^. Data was collected at a monochromator take-off angle of 90° using a wavelength of 2.41846(6) Å for high angular resolution. Orientations of the electric field with respect to the incident beam were measured by moving the ω-sample table in 15° steps (−57° ≤ ω ≤ 108°). Further details can be found elsewhere^43^,^44^.

For the *in situ* electric field TEM study the specimen was ultrasonically cut into a disc of 3 mm in diameter, mechanically thinned to a thickness of about 120 μm by tripod polishing and then dimpled to a final thickness of about 20 μm by Gatan Model 656 Dimple Grinder (Gatan Inc., United States). In order to remove stress induced by mechanical polishing the sample was annealed. Finally, Argon ion milling was performed (600 Dual Ion Mill, Gatan Inc., Unites States). Gold electrodes with a spacing of 100 μm were evaporated on top of the sample (Auto 306, Edwards Group Ltd, United Kingdom) and contacted with the modified *in situ* electric field holder Gatan 646 by using platinum wires (Gatan Inc., United States). The *in situ* TEM experiment was performed at a FEI CM20 instrument operated at 200 kV (FEI Corporation, The Netherlands). During the experiment the voltage was increased stepwise by 10 V. Maximum applied voltage was 400 V, which corresponds to an electric field of 4 kV/mm in the sample. Quantification of the strain output was performed by considering the grain elongation obtained from the mathematical subtraction of the virgin state as compared with the grain with presence of an electric field. The strain was calculated by comparing a total of 187 strain measurements along the subtracted image thus allowing to obtain a representative mean value for the whole grain.

## Additional Information

**How to cite this article**: Acosta, M. *et al*. Piezoelectricity and rotostriction through polar and non-polar coupled instabilities in bismuth-based piezoceramics. *Sci. Rep.* **6**, 28742; doi: 10.1038/srep28742 (2016).
